# The aggregation paradox for statistical rankings and nonparametric tests

**DOI:** 10.1371/journal.pone.0228627

**Published:** 2020-03-12

**Authors:** Haikady N. Nagaraja, Shane Sanders

**Affiliations:** 1 The Ohio State University, Division of Biostatistics, College of Public Health, Columbus, OH, United States of America; 2 Syracuse University, David B. Falk College of Sport & Human Dynamics, Syracuse, NY, United States of America; Roswell Park Cancer Institute, UNITED STATES

## Abstract

The relationship between social choice aggregation rules and non-parametric statistical tests has been established for several cases. An outstanding, general question at this intersection is whether there exists a non-parametric test that is *consistent upon aggregation* of data sets (not subject to *Yule-Simpson Aggregation Paradox* reversals for any ordinal data). *Inconsistency* has been shown for several non-parametric tests, where the property bears fundamentally upon robustness (ambiguity) of non-parametric test (social choice) results. Using the *binomial*(*n*, *p* = 0.5) random variable CDF, we prove that aggregation of *r*(≥2) constituent data sets—each rendering a qualitatively-equivalent *sign test for matched pairs* result—reinforces and strengthens constituent results (*sign test consistency*). Further, we prove that magnitude of *sign test consistency* strengthens in significance-level of constituent results (*strong-form consistency*). We then find preliminary evidence that *sign test consistency* is preserved for a generalized form of aggregation. Application data illustrate (*in*)consistency in non-parametric settings, and links with information aggregation mechanisms (as well as paradoxes thereof) are discussed.

## Introduction

The relationship between social choice aggregation rules and non-parametric statistical analysis is well-established [see, e.g., 1, 2, 3, 4, 5, 6, 7, 8, 9]. In a related literature, fundamental relationships between discrete choice and non-parametric statistical analysis have been developed [see 10, 11, 12, 13, 14, 15, 16, 17]. While violations of social choice principles can inform us as to the possible paradoxes present in non-parametric tests, the mapping is imperfect due to the issue of statistical significance. Consider a comoparison of three groups, where the term “group” refers to sampled elements of the same population. If, for example, a violation of transitivity occurs in a set of raw, ordinal data when comparing three groups pairwise, this does not imply that any sort of paradox is evident in the results of a corresponding set of pairwise significance tests. This distinction is studied prominently in Bargagliotti and Greenwell [9].

In a seminal work, Haunsperger and Saari [4] find that “…two or more data sets each may individually support a certain ordering of the samples under [the] *Kruskal-Wallis* [test], yet their union, or aggregate, yields a different outcome” [18, p. 261]. In other words, the *Kruskal-Wallis* (*KW*) test [19] is not necessarily *consistent upon aggregation* of data. The works of Haunsperger and Saari [4] and later of Haunsperger [18] provide characterizations as to aggregation paradoxes in non-parametric statistics. These works extend a prominent literature concerning the classic *Yule-Simpson* (association or statistical aggregation) *Paradox* [see 20 or 21 and, e.g., 22, 23, 24, 25, 26, 27, 28, or 29 for later applications] to the non-parametric realm. The *Yule-Simpson Paradox* has far-reaching implications within statistics and econometrics. Wardrop [25] demonstrates that the Paradox has an important bearing upon the existence (non-existence) of the hot-hand fallacy and related *law of small numbers*. Specifically, Wardrop notes that researchers studying whether a hot hand fallacy exists should analyze data at the level of aggregation that (potentially fallacy-prone) fans view the data [see, e.g., Miller and Sanjurjo [30] for recent, related work on the the hot hand fallacy].

Bickel et al. [31] find evidence that the Paradox can influence evaluation of gender bias in graduate admissions data. Specifically, statistically significant results at the program level need not hold when evaluating graduate admissions at the university level. Albers [32] finds a related result in evaluating gender bias in research funding. In light of the *Yule-Simpson Paradox*, these studies establish the importance of analyzing data at the appropriate level of aggregation, whatever it may be. Charig et al. [33] examine treatment effects for small and large kidney stones, both separately and pooled. Consistent with the *Paradox*, they find evidence that treatment efficacy reverses from separate to pooled tests.

Further developing the *Paradox* for non-parametric settings, Haunsperger [18] discovers a computable criterion by which the *consistency* (*upon aggregation*) of an ordinal data set (when *KW*-tested) can be characterized. Bargagliotti [34] finds that *Bhapkar’s V test* [35] and the *Wilcoxon-Mann-Whitney* (*WMW*) test [Wilcoxon 1945, Mann and Whitney 1947]—two other central non-parametric tests—are also not generally *consistent upon aggregation*. Hao and Houser [38] state, “Both robust and simple to implement, [the WMW test] has gained exceptional popularity among empirical scientists, even social scientists. For example, in 2009 almost half of papers in *Experimental Economics* used the *WMW* test. Hao and Houser [38] demonstrate that adaptive procedures for the *WMW* test can lead to “substantial improvements in the ability to detect differences in locations” [p. 1940]. Specifically, Gastwirth [39] and Hogg et al. [40] develop adaptive ranks tests with such a quality. In some respects, the *Yule-Simpson Paradox* relates to the power and sensitivity of given statistical tests, and how these qualities (may) relate to sample size. The *Paradox* also relates to a broader literature on information aggregation and paradoxes in which more information is not always revealed to result in better (expected) outcomes [see, e.g., Kaufmann and Weber [41], Koessler et al. [42], Bennouri et al. [43], Axelrod et al. [44], Hanson et al. [45]]. Of course, information aggregation paradoxes may be symptomatic of the decision-maker, of the information aggregation mechanism, or of both. Bennouri et al. [43] demonstrate that accepted information aggregation mechanisms can vary substantially in terms of effectiveness (i.e., in supporting optimal decisions).

The *KW test* [see, e.g., 46; 47; 48 for more recent descriptions and generalizations of the test] represents a non-parametric statistical analog of the *n*(≥2) group *rank sum* aggregation rule in social choice theory. The *WMW test* represents a related two-sample rank sum test. The *Wilcoxon rank sum test* and the *Mann-Whitney U test* are nominally distinct (rank sum) tests; their respective test statistics are linearly dependent upon one another such that the two tests lead to the same end (in terms of significance results). *Bhapkar’s V test*, on the other hand, compares categorically-assigned data across groups. That these non-parametric tests fail to attain general *consistency upon aggregation* is both surprising and problematic. Philosophically, these results are akin to a social aggregation rule that takes as inputs two *Yes* votes (e.g., from an election governed by a *Yes-No* voting rule), aggregates them, and returns *No* as the social choice (outcome). We might rather expect two such primitive data sets to strengthen the case for the original result upon aggregation. That such an expectation is not met for this set of tests suggests not only that aggregated statistical rankings are potentially arbitrary but that the primitive rankings—or any statistical rankings from the test—are themselves potentially arbitrary. That is, even a primitive data set is an aggregation of data subsets from the primitive set’s power set.

While Bargagliotti [34] shows a necessary condition (of the data) to ensure *consistent* (*upon aggregation*) results for the *KW* and *Bhapkar’s V tests*, respectively, the literature has not determined whether *inconsistency upon aggregation* is a general paradox among non-parametric statistical tests. Both the title of Haunsperger and Saari (“The Lack of Consistency for Statistical Decision Procedures”) and that of Haunsperger (“Aggregated Statistical Rankings are Arbitrary”) certainly leave open the intriguing possibility of a general result. Selvitella [49] further considers the “ubiquity” of the *Yule-Simpson Paradox*. Based upon early results within the literature, Haunsperger [18] writes, “Haunsperger and Saari [4] show that this Simpson-like paradox occurs with many, if not most, statistical decision procedures. Because of the results [therein], we must expect *KW* to be the non-parametric system most immune to such difficulties” [p. 263]. We consider, then, whether general *consistency under aggregation* is an *impossibility* among non-parametric tests. Alternative tests, such as the *sign test for matched pairs*, apply distinct aggregation rules to non-parametric data in order to assign aggregated statistical rankings of groups. Herein, we examine whether the *sign test for matched pairs* (heretofore, *“sign test”*) is generally *consistent upon aggregation*, where the *sign test* represents another central non-parametric test with a social choice aggregation rule analog. It is important to note that the *McNemar* [50] *test* is closely related to the *sign test for matched pairs*. Specifically, the latter test represents an exact test of the former.

The *sign test* uses a *binomial*(*n*, *p* = 0.5) test statistic to pairwise rank two groups from inter-group (inter-sample) comparisons of matched-pair elements. The *sign test* statistic is calculated in a manner that is procedurally-equivalent to the two-candidate case of *Borda rule* aggregation and to the two-candidate case of *majority rule* aggregation (the latter two of which are, then, themselves equivalent to one another in the two-group case). That is to say, each paired-element comparison of the *sign test* yields a mutually exclusive “vote” in favor of one of the two groups. This is equivalent to two-candidate *Borda* or two-candidate *majority rule* voting, whereby each voter makes a binary preference comparison of two candidates and (assuming voting is sincere) casts a mutually exclusive vote in favor of her preferred candidate. The two candidates are then ranked according to *majority rule* type aggregation.

In Section 2 of this paper, we construct a theoretical framework by which to consider whether the *sign test* is *consistent upon aggregation*. In Section 3, we utilize this framework to explore the properties of aggregated statistical rankings from the *sign test*. We ultimately find that statistical rankings from the *sign test* are *consistent upon aggregation*, and we present a general proof of the result. That is, we utilize the cumulative distribution function (cdf) of a *binomial*(*n*, *p* = 0.5) random variable to show generally that the aggregation of two or more data sets—each rendering a *sign test* result of the same quality—can only strengthen the original result (i.e., can only decrease the *p-value* of the *sign test* statistic). This result is shown for any number of aggregated (primitive) data sets. We prove an additional result as to the nature of *sign test consistency*. Namely, the *sign test p-value* exhibits a greater proportional decrease (in aggregation) as the original (primitive data *sign test*) *p-value* itself becomes smaller. In aggregation, then, we expect a greater proportional *p-value* decline given significant *sign test* results for the constituent, primitive data (than for insignificant *sign test* results). This represents something of a *strong form consistency* result, whereby data aggregation reinforces significant *sign test* results to a greater (proportional) degree. Lastly, we construct and define a generalized notion of aggregation to consider the aggregation of data sets of different sample sizes. We find preliminary evidence that the *sign test* is also *consistent upon generalized aggregation*. Analysis of the generalized form is of great practical importance, as data sets of different sample sizes are often aggregated (e.g., in the case of many longitudinal and unbalanced panel data sets).

The matched pairs nature of the *sign test* is crucial to the result of *consistency upon aggregation*. In the aggregation of ordinal or numerical data with pre-assigned matched pairs, data are not aggregated into an overall outcome sequence but, rather, into a larger set of matched pairs. As such, *sign test* aggregation is reinforcing or *consistent* rather than potentially *inconsistent*. We find that it is possible for a non-parametric test to exhibit *consistency*.

In Section 4, we develop a generic application in which we present a set of ordinal data for two distinct groups. Elements of the data can be paired across group (based, e.g., on some characteristic such as “twin-ness” in a control-treatment study) and analyzed via the *sign test* (for matched pairs). Alternatively, elements for the respective groups may remain unpaired, and elements can then be aggregated into an outcome or rank-sequence such that overall group rankings are generated from a *WMW rank sum test*. Within the application, we demonstrate a case of *inconsistency upon aggregation* for the *WMW test*. We do so to show readers possible characteristics of an ordinal data that is susceptible to *inconsistency*. For the same ordinal data, we demonstrate an example of the (proved, general) *consistency upon aggregation* of the *sign test*. As such, the procedural differences between the two tests are also highlighted (with respect to the *consistency* property). For the application, we aggregate sets of ordinal data twice, thrice, and four times. It is shown that the *sign test p-value* shrinks monotonically in the number of data sets aggregated. This follows from the general results of Section 3. For the same ordinal data, we observe a case in which the *WMW test p-value* rises monotonically in the number of data sets aggregated (as was shown to be possible by Bargagliotti [34]).

Section 5 identifies and develops an empirical application from the sport of high school team cross country running, a sport that uses rank sum scoring to map from an individual outcome sequence (of individual finishing positions) to team scores. The empirical application uncovers evidence of *rank sum* “*inconsistency*” and *sign test* *consistency* for high school team cross country meet data. Section 6 concludes.

## 2 Theoretical set-up and preliminary Lemmas

### 2.1 Preliminary definitions

Let us begin our exposition with some definitions concerning data aggregation.

**Definition of *n-aggregation* of data**: Consider a primitive, *n*-element data set, *A*. Let *n-aggregation* involving *A* be an aggregation of *A* and *r* − 1 ($r\in Z^{+}$) other data set(s) each of sample size *n* to form an aggregated data set.

Haunsperger and Saari [4] and Haunsperger [18] construct a powerful methodology to assess *consistency upon aggregation* for *KW*. Namely, they find a condition on a matrix of ordinal data rankings that is equivalent to *consistency* (mutually exclusive with *inconsistency*). As they utilize a matrix approach, their methodology considers balanced sample size data aggregation (i.e., *n-aggregation*). Note that *n-aggregation* is a restricted form of data aggregation, as it does not consider the aggregation of two or more data sets of different sample sizes. The non-parametric aggregation paradox has focused on this restricted form of data aggregation to date (see, e.g., any of the seminal papers mentioned in the introduction). This is perhaps due to the tractability of examining this form of aggregation, as we will observe in the present analysis. In this study, our primary theorems will concern *n*-aggregation of ordinal data. However, we also present preliminary results for a more general version of data aggregation of the following form.

**Definition of n_i_-*aggregation* of data**: Consider a primitive, *n*_0_-element data set, *A*. Let *n_i_-aggregation* involving *A* be an aggregation of *A* and *r* − 1 ($r\in Z^{+}$) other data set(s) of respective sample sizes *n*_1_, *n*_2_, …, *n*_*r*−1_ to form an aggregated data set.

For tractability, *n-aggregation* has been incorporated as a standard approach in examining the *Yule-Simpson Paradox* for ordinal data. However, analysis of the generalized *n*_*i*_-aggregation form is an important consideration. Often, data sets of different sample sizes are pooled (e.g., in the case of many longitudinal and unbalanced panel data sets).

Let us now define *consistency upon aggregation* largely as in Haunsperger [18]. She states, “A statistical procedure that endows a set of data with an ordinal ranking of the candidates is consistent [upon] aggregation if the aggregate of any [*r*] sets of data, each of which corresponds to a given ordering of the candidates, gives rise to the same ordering of the candidates for any positive integer [*r*]” [p. 264]. We refine the definition slightly to incorporate the notion of statistical significance explicitly as follows.

**Definition of *Consistency upon Aggregation***: A statistical procedure that endows a set of data with an ordinal ranking of the candidates is *consistent upon aggregation* if the aggregate of any *r* sets of data, each of which corresponds to a given statistically-significant ordering of the candidates, gives rise to the same statistically-significant ordering (i.e., at the same *α*-level) of the candidates for any positive integer *r* and for all possible *α* levels.

Thinking of *consistency* from the perspective of significance testing, we can evaluate the *consistency* of a procedure by simply verifying whether it is possible for the *p-value* of a test result to rise following aggregation. If so, statistically-significant strict orderings can be lost in aggregation such that the procedure is not generally *consistent*.

### 2.2 Theoretical setup

Let *X*_*n*_ be a *Binomial* random variable with parameters *n* and *p* = 1/2, and *N* = *rn* for some integer *r* > 1. Then, the *sign test* for matched pairs is *consistent upon n-aggregation* if and only if (iff) for any *k* between *n*/2 and *n*,

$$\begin{array}{c} P\left(X_{N}\geq rk\right)<P\left(X_{n}\geq k\right). \end{array}$$ (1)

From (1), it may appear that we are aggregating primitive data sets each of which renders the very same test statistic value, *k*. In fact, (1) provides a general condition for *consistency upon aggregation*. Let us think of *k* as the lowest *binomial*(*n*, *p* = 0.5) test statistic value (from tests of given sets of primitive data) such that a given statistically-significant ordering emerges from the primitive data (i.e., at a stipulated *α*-level). As such, the term *rk* represents a lower bound on the aggregated data test statistic value, and the left-hand side *p-value* in (1) represents an upper-bound for the *p-value* from a test of the aggregated data. Then, (1) states that the *p-value* for an associated test of the aggregated data will always be less than the highest (but still significant) *p-value* among the associated primitive data sets.

To establish (1), we first define

$$\begin{array}{c} a_{j}=\sum_{i=0}^{r-1}a_{j}\left(i\right),\;b_{j}=(\begin{array}{c} n\\ j \end{array}),c_{j}=\frac{a_{j}}{b_{j}},\;\text{and}\;d_{j}=\frac{\sum_{i=j}^{n}a_{i}}{\sum_{i=j}^{n}b_{i}}, \end{array}$$ (2)

where

$$\begin{array}{c} a_{j}\left(i\right)=(\begin{array}{c} rn\\ rj+i \end{array}),i=0,\ldots ,\left(r-1\right), \end{array}$$ (3)

and (*n* − 1)/2 < *k* ≤ *j* ≤ *n*. Here, *a*_*j*_ counts the distinct number of ways of obtaining between *rj* and *rj* + *r* − 1 successes in *rn* Bernoulli trials, and *b*_*j*_ counts the number of ways of obtaining *j* successes in *n* Bernoulli trials. The coefficients *c*_*j*_ and *d*_*j*_ are useful for studying the relative variation in *a*_*j*_ and *b*_*j*_ and their upper tail partial sums. We now show that *c*_*j*_ and consequently *d*_*j*_ are strictly increasing in the range of our interest.

**Lemma 1** *For sequences a_j_, b_j_, c_j_ given in* (2), *and a_j_(i) given in* (3), *the following properties hold*.

(a) *For a fixed i* > 0, *a_j_*(*i*)/*b_j_ strictly decreases as j increases in the interval* [((*n* − 1)/2) − (*i*/*r*), *n*].
(b) *a_j_*(0)/*b_j_ strictly decreases as j increases in the interval* ((*n* − 1)/2, *n*], *and when j* = (*n* − 1)/2 *is an integer, a_j_*(0)/*b_j_* = *a*_*j*+1_(0)/*b*_*j*+1_.
(c) *c_j_* = *a_j_/b_j_ strictly decreases for j in the interval* [(*n* − 1)/2, *n*].

**Proof**. From the expressions for *a*_*j*_(*i*) and *b*_*j*_ given in (3) and (2) respectively, we note that

$$\begin{array}{c} \frac{a_{j}\left(i\right)}{b_{j}}=\frac{(rn)!j!(n-j)!}{(rj+i)!(rn-rj-i)!n!}\;\;\text{and}\;\;\frac{a_{j+1}\left(i\right)}{b_{j+1}}=\frac{(rn)!(j+1)!(n-j-1)!}{(rj+r+i)!(rn-rj-r-i)!n!}. \end{array}$$

Canceling out the common factors in the above two expressions, we conclude that the condition *a*_*j*_(*i*)/*b*_*j*_ > *a*_*j*+1_(*i*)/*b*_*j*+1_ holds iff

$$\begin{array}{c} \frac{\left(n-j)(r(j+1)+i\right)}{\left(j+1)(r(n-j)-i\right)}\prod_{t=1}^{r-1}\frac{r(j+1)+i-t}{r(n-j)-i-t}>1, \end{array}$$ (4)

for any *j* ≤ (*n* − 1) in the specified range. The first factor on the left side of (4) above exceeds 1 iff (*n* − *j*)*i* > −*i*(*j* + 1). For any *j*, this is true for all *i* > 0, and when *i* = 0, the first factor equals 1. The ratio corresponding to the *t*th factor in the product exceeds 1 iff *r*(*j* + 1) + *i* − *t* > *r*(*n* − *j*) − *i* − *t*, or equivalently *j* > (*n* − 1)/2 − *i*/*r*. When *j* = (*n* − 1)/2 − *i*/*r* is an integer, the product equals 1, and for *i* > 0 the first factor exceeds 1 and so the strict monotonicity of *a*_*j*_(*i*)/*b*_*j*_ holds in [(*n* − 1)/2 − *i*/*r*, *n*]; thus the conclusion in part (a) is established.

When *j* = (*n* − 1)/2 is an integer, the first factor as well as the product term equal 1, leading us to the conclusion in part (b), and strict monotonicity holds for *j* > (*n* − 1)/2.

Since *a*_*j*_ is the sum of the *a*_*j*_(*i*), and as long as at least one of the *a*_*j*_(*i*)/*b*_*j*_ shows strict monotonicity in *j*, the strict monotonicity of *c*_*j*_ follows. This claim holds for an integer *j* = (*n* − 1)/2 also, completing the proof of part (c).

**Lemma 2** *For the d_j_ defined in* (2),

$$\begin{array}{c} d_{n}<d_{n-1}<\cdots <d_{\frac{n}{2}}\;for\;even\;n; \end{array}$$

$$\begin{array}{c} d_{n}<d_{n-1}<\cdots <d_{\frac{n+1}{2}}<d_{\frac{n-1}{2}}\;for\;odd\;n. \end{array}$$

**Proof**. We use an induction argument. For this, first we prove that *d*_*n*_ < *d*_*n*−1_. Recall that *d*_*n*_ = *a*_*n*_/*b*_*n*_ and *d*_*n*−1_ = (*a*_*n*_ + *a*_*n*−1_)/(*b*_*n*_ + *b*_*n*−1_), where the *a*_*j*_ and *b*_*j*_ are defined in (2). Hence, the condition: *d*_*n*_ < *d*_*n*−1_ is equivalent to the condition:

$$\begin{array}{c} \frac{a_{n}}{b_{n}}<\frac{a_{n}+a_{n-1}}{b_{n}+b_{n-1}}. \end{array}$$ (5)

Since denominators above are positive, (5) holds iff *a*_*n*_ *b*_*n*_ + *a*_*n*_ *b*_*n*−1_ < *a*_*n*_ *b*_*n*_ + *a*_*n*−1_ *b*_*n*_, or equivalently,

$$\begin{array}{c} \frac{a_{n}}{b_{n}}<\frac{a_{n-1}}{b_{n-1}}. \end{array}$$

This condition follows from part (c) of Lemma 1 since *c*_*j*_ = *a*_*j*_/*b*_*j*_.

Next, assuming *d*_*j*+1_ < *d*_*j*_, we will prove that *d*_*j*_ < *d*_*j*−1_. From the definition of *d*_*j*_ given in (2), we conclude that

$$\begin{array}{ccc} d_{j+1}<d_{j} & \Leftrightarrow & \frac{\sum_{i=j+1}^{n}a_{i}}{\sum_{i=j+1}^{n}b_{i}}<\frac{\sum_{i=j}^{n}a_{i}}{\sum_{i=j}^{n}b_{i}}\\ & \Leftrightarrow & b_{j}\sum_{i=j+1}^{n}a_{i}<a_{j}\sum_{i=j+1}^{n}b_{i}, \end{array}$$ (6)

upon cross-multiplication and cancellation of common terms. Similarly, the condition: *d*_*j*_ < *d*_*j*−1_ is equivalent to the condition:

$$\begin{array}{c} b_{j-1}\sum_{i=j}^{n}a_{i}<a_{j-1}\sum_{i=j}^{n}b_{i}. \end{array}$$ (7)

The left side sum in (7) is

$$\begin{array}{c} b_{j-1}a_{j}+b_{j-1}\sum_{i=j+1}^{n}a_{i}=b_{j-1}a_{j}+\frac{b_{j-1}}{b_{j}}\;b_{j}\sum_{i=j+1}^{n}a_{i}<b_{j-1}a_{j}+\frac{b_{j-1}}{b_{j}}\;a_{j}\sum_{i=j+1}^{n}b_{i}, \end{array}$$ (8)

where the inequality above follows from (6). From part (c) of Lemma 1, it follows that *c*_*j*_ < *c*_*j*−1_; that is, (*a*_*j*_/*b*_*j*_)<(*a*_*j*−1_/*b*_*j*−1_) or *a*_*j*_ *b*_*j*−1_ < *a*_*j*−1_ *b*_*j*_. Hence, the upper bound in (8),

$$\begin{array}{c} b_{j-1}a_{j}+\frac{b_{j-1}}{b_{j}}\;a_{j}\sum_{i=j+1}^{n}b_{i}<b_{j}a_{j-1}+\frac{b_{j}a_{j-1}}{b_{j}}\;\sum_{i=j+1}^{n}b_{i}=a_{j-1}\sum_{i=j}^{n}b_{i}; \end{array}$$

this is the right side of the (strict) inequality in (7). This strict inequality (in (7)) also holds for *j* − 1 = (*n* − 1)/2 for odd *n*, and *j* − 1 = *n*/2 for even *n*. This establishes our claim.

**Remark 1**. This monotonicity property of the ratio of the partial sums is perhaps a well-known result in the “Inequalities” literature. We have not found a convenient reference, and have chosen to prove it with elementary principles; we have also fine-tuned the result for the even *n* case.

## 3 Main results

**Theorem 1 *Consistency upon Aggregation***: *Let X_n_ be a Binomial random variable with parameters n and p* = 1/2. *Then, with N* = *rn, the inequality in* (1) *holds for all integers k* ≥ *n*/2, *and integers r* ≥ 2.

**Proof**. In terms of the coefficients introduced in (2) and (3),

$$\begin{array}{c} P\left(X_{N}\geq rk\right)=2^{N}\sum_{j=k}^{n}a_{j} \end{array}$$

and

$$\begin{array}{c} P\left(X_{n}\geq k\right)=2^{n}\sum_{j=k}^{n}b_{j}, \end{array}$$

where *N* = *rn*. The inequality in (1) can be expressed as

$$\begin{array}{c} \frac{P(X_{N}\geq rk)}{P(X_{n}\geq k)}\equiv {\left(\frac{1}{2}\right)}^{(r-1)n}d_{k}\;\;<\;\;1. \end{array}$$ (9)

We have seen in Lemma 2 that *d*_*k*_ strictly increases as *k* decreases toward *n*/2. Thus, the maximum value for the ratio of the two binomial probabilities in (9) is attained with *k* = (*n* + 1)/2 for odd *n* and *k* = *n*/2 for even *n*. We now show that this maximum value is less than 1 for both odd and even *n*.

When *n* is odd and *n* = 2*m* − 1, we take *k* = *m* = (*n* + 1)/2 in (9). By the symmetry of the binomial probabilities, *P*(*X*_*n*_ ≥ *m*) = 1/2.

Now *N*(= *rn*) may be even or odd. When *N* is even, *r*(≥2) is also even, and *rn*/2 is the median of *X*_*N*_. Further, *P*(*X*_*N*_ ≥ *rn*/2)>1/2 and *P*(*X*_*N*_ ≥ *rn*/2 + 1)<1/2. Thus,

$$\begin{array}{c} P\left(X_{N}\geq rm\right)=P\left(X_{N}\geq \frac{r(n+1)}{2}\right)\leq P\left(X_{N}\geq \frac{rn}{2}+1\right)<\frac{1}{2}, \end{array}$$

where the first inequality holds since *r*/2 ≥ 1. When *N*(= *rn*) is odd, *r*(≥3) is also odd, and *P*(*X*_*N*_ ≥ (*rn* + 1)/2) = 1/2. Thus,

$$\begin{array}{c} P\left(X_{N}\geq rm\right)=P\left(X_{N}\geq \frac{r(n+1)}{2}\right)<P\left(X_{N}\geq \frac{rn+1}{2}\right)=\frac{1}{2}, \end{array}$$

where the strict inequality holds since (*rn* + *r*)/2 and (*rn* + 1)/2 are both integers and the difference (*r* − 1)/2 ≥ 1. Thus, the strict inequality in (9) holds for all *k* ≥ *n*/2 when *n* is odd.

When *n* is even and *n* = 2*m*, we take *k* = *n*/2 = *m*, and note that *N* is always even. By the symmetry of the binomial probabilities,

$$\begin{array}{c} P\left(X_{n}\geq m\right)=\frac{1}{2}+\frac{1}{2}P\left(X_{n}=m\right); \end{array}$$

$$\begin{array}{c} P\left(X_{N}\geq rm\right)=\frac{1}{2}+\frac{1}{2}P\left(X_{N}=rm\right). \end{array}$$

Thus, the strict inequality in (9) holds iff

$$\begin{array}{c} P\left(X_{N}=rm\right)<P\left(X_{n}=m\right). \end{array}$$ (10)

We will now establish (10). Note that *X*_*N*_ has the same distribution as *X*_*n*_ + *Y* where *Y* is Binomial with parameters *N* − *n* and *p* = 1/2, and *X*_*n*_ and *Y* are independent.

$$\begin{array}{c} P\left(X_{N}=rm\right)=\sum_{i=0}^{n}P\left(X_{n}=i\right)P\left(Y=rm-i\right). \end{array}$$

Since $\left(\begin{array}{c} n\\ i \end{array}\right)$ is largest when *i* = *n*/2 = *m*, so is *P*(*X*_*n*_ = *i*). Hence,

$$\begin{array}{c} P\left(X_{N}=rm\right)<P\left(X_{n}=m\right)\sum_{i=0}^{n}P\left(Y=rm-i\right)\leq P\left(X_{n}=m\right), \end{array}$$

which establishes (10). That is, (9) holds for all *k* ≥ *n*/2 when *n* is even as well.

A similar result on the lower tail probabilities can be established by symmetry. As such, we have shown that any significant sign test result is *consistent upon n-aggregation*. This result has important implications for classic results (e.g., by [31] and [33]). If we apply the sign test to the underlying data of these studies, while normalizing the sample size to equality across group and across treatment, it is not possible for an aggregation paradox to occur. Later in the paper, we will consider whether the sign test is susceptible to instances of aggregation paradox given unequal sample sizes across group. We can refine Theorem 1 by observing that we have proved a great deal more. In fact, we have established the following.

**Theorem 2 *Strong Form Consistency upon Aggregation***: *Let X_N_ be a Binomial random variable with parameters N* = *rn and p* = 1/2 *where n* ≥ 1 *and r* ≥ 2 *is an integer. Then, as the integer k decreases in* [*n*/2, *n*], *P*(*X_N_* ≥ *rk*)/*P(X_n_* ≥ *k*) *monotonically increases with a strict upper bound of* 1.

That the ratio is bounded by 1 follows from Theorem 1. Theorem 2 tells us that the *n-aggregated* data associated *p-value* rises toward the primitive data associated *p-value* as the primitive data *p-value* rises. In other words, *n-aggregation* creates a greater proportional decrease in the *sign test p-value* as the original (primitive) *p-value* itself becomes smaller. In aggregation, then, we expect a greater proportional *p-value* decline given significant *sign test* results for the constituent, primitive data (than for insignificant *sign test* results). This represents something of a *strong form consistency* result, whereby data aggregation reinforces significant *sign test* results to a greater (proportional) degree. A more general result of the following form is highly desirable.

**Theorem 3 (Conjecture)** *Let X_N_ denote a Binomial random variable with parameters N and p* = 1/2. *Then for positive integers n*_1_ < *n*_2_ *and constant c* ∈ [1/2, 1] *such that cn*_1_ *and cn*_2_ *are integers, the following inequality holds*.

$$\begin{array}{c} P(\frac{X_{n_{2}}}{n_{2}}\geq c)<P(\frac{X_{n_{1}}}{n_{1}}\geq c). \end{array}$$ (11)

We are able to establish this claim for the boundary values of *c* (that is, *c* = 1/2 or 1) using the arguments presented earlier, and conjecture that the result is true for other *c* values in [1/2, 1]. Limited computational work supports this conjecture. Further, a large-sample approximation, described below, leads us to believe in the conjecture, especially when *n*_1_ and *n*_2_ are large and far apart.

From central limit theorem, as *n* → ∞, we know that $\sqrt{n}\left(X_{n}/n-1/2\right)\overset{d}{\to }N\left(0,1/4\right)$. Further, the binomial distribution, being already unimodal and symmetric (as *p* = 1/2), the convergence is fast. So, if *n* is large we have the following approximation:

$$\begin{array}{c} P(\frac{X_{n}}{n}\geq c)=P(\sqrt{n}\left(\frac{X_{n}}{n}-\frac{1}{2}\right)\geq \sqrt{n}\left(c-\frac{1}{2}\right))\approx 1-\Phi (\sqrt{4n}\left(c-\frac{1}{2}\right)), \end{array}$$ (12)

where Φ is the cdf of a standard normal random variable. This “close” approximation to the tail probability strictly decreases as *n* increases as long as *c* > 1/2 and hence we believe (11) holds for large *n*_1_ < *n*_2_.

**Remark 2**. When *c* = 1/2 and *n*_1_ and *n*_2_ are odd integers, the strict inequality claimed in (11) does not hold and each of the probabilities equals 1/2 as for an odd integer *n*,

$$\begin{array}{c} P\left(X_{n}\geq n/2\right)=P\left(X_{n}\geq \left(n+1\right)/2\right)=1/2. \end{array}$$

Also, it follows from (12) that, irrespective of the odd or even nature of *n*, the limiting value of this probability is 1/2.

## 4 Application 1: Demonstrating aggregation of statistical rankings for *Sign* and *WMW tests*

### 4.1 Tests of a primitive, ordinal data set

Consider two groups, *A* and *B*. Each group consists of nine elements or data points (i.e., $\frac{n}{2}=9$ or *n* = 18). That is, *A* = {*a*_1_, *a*_2_, …, *a*_9_} and *B* = {*b*_1_, *b*_2_, …, *b*_9_} such that the primitive (unaggregated) data for the *WMW rank sum test* set-up is an outcome sequence of 18 ordinal (rank-ordered) data points. For the application, let

$$\begin{array}{c} F_{AB}=a_{1},a_{2},a_{3},a_{4},a_{5},a_{6},a_{7},a_{8},b_{1},b_{2},b_{3},b_{4},b_{5},b_{6},b_{7},b_{5},b_{6},b_{7},b_{8},b_{9},a_{9} \end{array}$$

represent the ordinal data (sequence). For the *sign test* set-up, each *a*_*i*_ ∈ *A* is matched with a counterpart *b*_*j*_ ∈ *B* according to the matching criterion. As we are *a priori* unsure (notationally) which *b*_*j*_ will be matched with each given *a*_*i*_, we say that each *a*_*i*_ is matched with some *b*_*π*(*i*)_, where *π*: *D* → *D* is defined as a bijection between *D* = {1, 2, 3, …, 8, 9} and itself such that element *a*_*i*_ ∈ *A* and element *b*_*π*(*i*)_ ∈ *B* uniquely pair with one another for all *i* ∈ *D*. As such, the following matched pairs data set is generated (uniquely in the present example) from the outcome sequence *F*_*AB*_:

$$\begin{array}{c} M_{AB}=\left\{\left(a_{1},b_{\pi (1)}\right),\left(a_{2},b_{\pi (2)}\right),\left(a_{3},b_{\pi (3)}\right),...,\left(b_{\pi (9)},a_{9}\right)\right\}. \end{array}$$

The set *M*_*AB*_ is a set of rank-ordered (matched) pairs of elementary data points. That is to say, *M*_*AB*_ is a set whose elements are outcome sequences on matched pairs. If an element of *M*_*AB*_ is represented as (*a*_*i*_, *b*_*j*_), this is equivalent to finding that *a*_*i*_ ≻ *b*_*j*_ (“*a*_*i*_ ranks higher than *b*_*j*_”) in outcome sequence *F*. If an element of *M*_*AB*_ is represented as (*b*_*j*_, *a*_*i*_), this is equivalent to saying that *b*_*j*_ ≻ *a*_*i*_ in outcome sequence *F*_*AB*_. As such, *F*_*AB*_ and *M*_*AB*_ are different representations of the same ordinal data set. Whereas *F*_*AB*_ is a sequence representation of the data, *M*_*AB*_ is a matched-pairs representation. For the application data—but not generally—*M*_*AB*_ is uniquely derived from *F*_*AB*_. That is to say, the elements of *F*_*AB*_ are sequenced such that the ordering of any given matched pair in *F*_*AB*_ is invariant to the specific match formed. That is, when *i* ∈ {1, 2, 3, .., 8}, we know that *a*_*i*_ ≻ *b*_*j*_ regardless of which *j* is paired with *i*. When *i* = 9, we know that *b*_*j*_ ≻ *a*_*i*_ regardless of which *j* is paired with *i*. For the application data (but not generally), one can uniquely derive *M*_*AB*_ from *F*_*AB*_. However, it is never possible to derive *F*_*AB*_ uniquely from *M*_*AB*_. That is to say, *F*_*AB*_ sometimes maps to a unique *M*_*AB*_. Given only *M*_*AB*_, however, one can never map to a unique *F*_*AB*_. In such a case, one is always missing some rank comparison information needed to support a super-ordering or outcome sequence of the overall data. Specifically, one cannot obtain rank comparisons of unmatched elements (*a*_*i*_ and *b*_*π*(*j*)_, *j* ≠ *i*) from *M*_*AB*_.

From *F*_*AB*_, we can calculate rank sum scores for *A* and *B*, $R_{F_{AB}}\left(A\right)$ and $R_{F_{AB}}\left(B\right)$. Generally, we define $R_{F_{XY}}\left(.\right)$ as follows. Consider two groups, *X* and *Y*, and let *F*_*XY*_ represent an outcome sequence between elements of the two groups. For each element *x*_*i*_ ∈ *X*, let $x_{i}^{+}=\left\{a\in F_{XY}|a≻x_{i}\right\}$. Then, the rank of *x*_*i*_ in the sequence *F*_*XY*_, *r*(*x*_*i*_|*F*_*XY*_), equals $|x_{i}^{+}|+1$, and the rank sum score for group *X* given *F*_*XY*_ is represented as $R_{F_{XY}}\left(X\right)=\Sigma_{x_{j}\in X}\;r\left(x_{j}|F_{XY}\right).$ Given the outcome sequence *F*_*AB*_ specified earlier in this application, then, we obtain

$$\begin{array}{c} R_{F_{AB}}\left(A\right)=1+2+3+4+5+6+7+8+18=54;R_{F_{AB}}\left(B\right)=\left(18\times 19/2\right)-54=117, \end{array}$$

and apply the *WMW rank sum test*. Let $R_{F_{AB}}=\max\left\{R_{F_{AB}}\left(A\right),R_{F_{AB}}\left(B\right)\right\}$. Then, the upper-tail *WMW rank sum p-value* for this sample difference, $P(R_{F_{AB}}\geq 117|n=18)$, is equal to 0.0027. Then, we conclude from a *WMW test* of the primitive data that *A*≻_*α* = 0.01_ *B*, where the notation “≻_*α* = 0.01_” reads “ranks significantly higher than at the *α* = 0.01 significance level.” In other words, *A* has a significantly lower rank sum score than *B* such that we conclude from *F*_*AB*_ that group *A* ranks significantly higher than group *B* at the assigned significance level.

Let us consider the same data in matched pairs form, *M*_*AB*_. Of the 9 matched pairs, an element of *A* outranks the corresponding element of *B* for 8 of 9 matched pairs. Formally, let us define the *sign test* statistic value for *A*, $S_{M_{AB}}\left(A\right)$, as the number of elements *a*_*i*_∈ *A* such that *a*_*i*_≻ $b_{\pi_{i}}$: $S_{M_{AB}}\left(A\right)=\left|\left\{a_{i}\in A:a_{i}≻b_{\pi_{i}}\right\}\right|$. Therefore, we obtain the *sign test* values for the primitive data as $S_{M_{AB}}\left(A\right)=8$ and $S_{M_{AB}}\left(B\right)=1$ and let $S_{M_{AB}}=\max\left\{S_{M_{AB}}\left(A\right),S_{M_{AB}}\left(B\right)\right\}.$

The upper-tail *sign test* for matched pairs *p-value* for this sample difference, $P(S_{M_{AB}}\geq 8\;|\;\frac{n}{2}=9)$ is equal to 0.0098. Then, we conclude from a *sign test* for matched pairs of the primitive data that *A* ≻_*α* = 0.01_ *B*. For this primitive application data, the *WMW test* and the *sign test* each provide corresponding results at all standard significance levels.

### 4.2 Tests of aggregated, ordinal data

Now, let us replicate the (ordinality of the) primitive data once, combine the primitive data and its ordinal replicate, and re-test the aggregated data. One should note that an ordinally-replicated data set may be quite different numerically from the primitive (source) data from which it arises such that two ordinally-identical data sets can combine to form various, feasible aggregated sequences. The aggregated outcome sequence consists of 36 rank-ordered elements. One possible aggregated outcome sequence is:

$$\begin{array}{c} FF_{AB}^{\prime }=a_{1},a_{2},a_{3},a_{4},a_{5},a_{6},a_{7},a_{8},b_{1},b_{2},b_{3},b_{4},b_{5},b_{6},b_{7},b_{5},b_{6},b_{7},b_{8},b_{9},F_{AB}^{\prime },a_{9} \end{array}$$

where $F_{AB}^{\prime }=a_{1}^{\prime },a_{2}^{\prime },a_{3}^{\prime },a_{4}^{\prime },a_{5}^{\prime },a_{6}^{\prime },a_{7}^{\prime },a_{8}^{\prime },b_{1}^{\prime },b_{2}^{\prime },b_{3}^{\prime },b_{4}^{\prime },b_{5}^{\prime },b_{6}^{\prime },b_{7}^{\prime },b_{5}^{\prime },b_{6}^{\prime },b_{7}^{\prime },b_{8}^{\prime },b_{9}^{\prime },a_{9}^{\prime }$ is ordinally equivalent to *F*_*AB*_. In this aggregated sequence, we have simply spliced the replicate sequence, $F_{AB}^{\prime }$, into *F*_*AB*_ such that $F_{AB}^{\prime }$ is strung (consecutively) between the penultimate and ultimate elements of the original sequence. The implication of this aggregated outcome sequence is that—for the most part—the numerical values of the original data outrank those of the ordinal-replicate data (despite the identical ordinal quality of the two data sets). From $FF_{AB}^{\prime }$, we find that

$$\begin{array}{c} R_{FF_{AB}^{\prime }}\left(A\right)=1+2+3+4+5+6+7+8+18+19+20+21+22+23+24+25+35+36=279; \end{array}$$

$$\begin{array}{c} R_{FF_{AB}^{\prime }}\left(B\right)=\left(36\times 37/2\right)-279=387. \end{array}$$

The upper-tail *WMW rank sum p-value* for this sample difference, $P(R_{FF_{AB}^{\prime }}\geq 387\;|\;N=2n=36)$, is equal to 0.0438. At the *α* = 0.01 significance level, then, we conclude from a *WMW test* of the aggregated data that *A*∼_*α* = 0.01_ *B* (i.e., that *A* and *B* are rank-indistinguishable at the *α* = 0.01 significance level), where “∼_*α* = 0.01_” reads “is rank-indistinguishable to at the *α* = 0.01 significance level.”

We now consider a *sign test* for matched pairs upon the same aggregated data. Specifically, we combine *M*_*AB*_ and its replicate into the same data set to obtain the set $MM_{AB}^{\prime }$,

$$\{\left(a_{1},b_{\pi (1)}\right),\left(a_{2},b_{\pi (2)}\right),\left(a_{3},b_{\pi (3)}\right),...,\left(b_{\pi (9)},a_{9}\right),\left(a_{1}^{\prime },b_{\pi (1)}^{\prime }\right),\left(a_{2}^{\prime },b_{\pi (2)}^{\prime }\right),\left(a_{3}^{\prime },b_{\pi (3)}^{\prime }\right),...,\left(b_{\pi (9)}^{\prime },a_{9}^{\prime }\right)\}.$$

As *M*_*AB*_ is an unordered set of matched pairs (i.e., the set of elements is unordered, whereas each element is itself an ordered pair), the aggregation of *M*_*AB*_ and its ordinal replicate, $M_{AB}^{\prime }$, is also an unordered set of matched pairs. As such, $MM_{AB}^{\prime }$ features the same matched pair ordinal comparisons as does *M*_*AB*_, while representing each such pair twice. Matches for the *sign test* are pre-assigned at the primitive data level according to the (also pre-assigned) matching criterion (e.g., “twinness”). Therefore, matched pairings are preserved in aggregation. This can be said in a more straightforward manner: The identity of one’s twin (match), once assigned, is invariant to the level of data aggregation. Matched pairing preservation in data aggregation is an important property that causes the *sign test* to be distinct from independent sample non-parametric tests. As the profile of matched pairings is itself a feature of the primitive data in the case of the *sign test* (in much the same way that the primitive data outcome sequence is a feature of the primitive data in the case of a *WMW test*), one would not be strictly aggregating the primitive data if one were to permute the matched pairings in aggregation. Rather, one would thereby change a feature of the primitive data at the same time (in the same manner that a re-ordering of primitive data would go beyond aggregation in the case of a *WMW test*). Given this feature of the *sign test*, the matched pair elements of *M*_*AB*_ and $M_{AB}^{\prime }$, respectively, are not rank-compared with one another (across primitive group) within a sign test analysis of $MM_{AB}^{\prime }$.

Of the eighteen matched pairs in the once ordinally-replicated data, an element of *A* outranks the corresponding element of *B* for sixteen matched pairs. Therefore, we have the followingsign-test statistic for the aggregated data:

$$\begin{array}{c} S_{MM_{AB}^{\prime }}\left(A\right)=16\;S_{MM_{AB}^{\prime }}\left(B\right)=2. \end{array}$$

Then, the upper-tailed *sign test* for matched pairs *p-value* for this test statistic value, $P(S_{MM_{AB}^{\prime }}\geq 16)$ is equal to 0.000484. Then, we conclude from a *sign test* for matched pairs of the aggregated data that *A* ≻_*α* = 0.01_ *B*. For the once ordinally-replicated data, the *sign test* retains significance at the *α* = 0.01 level. Consistent with our general results, the *sign test p-value* diminishes with data aggregation in this case. On the other hand, the *WMW test* loses significance at the *α* = 0.01 level when applied to the once ordinally-replicated data.

To consider the result of Theorem 2, we alter *M*_*AB*_ as follows.

$$\begin{array}{c} {\tilde{M}}_{AB}=\left\{\left({\tilde{a}}_{1},{\tilde{b}}_{\pi (1)}\right),\left({\tilde{a}}_{2},{\tilde{b}}_{\pi (2)}\right),{\tilde{(a}}_{3},{\tilde{b}}_{\pi (3)}\right),...,\left({\tilde{b}}_{\pi (8)},{\tilde{a}}_{8}\right),\left({\tilde{b}}_{\pi (9)},{\tilde{a}}_{9}\right)\}. \end{array}$$

We also alter $M_{AB}^{\prime }$ as follows.

$$\begin{array}{c} {\tilde{M}}_{AB}^{\prime }=\left\{\left({\tilde{a}}_{1}^{\prime },{\tilde{b}}_{\pi (1)}^{\prime }\right),\left({\tilde{a}}_{2}^{\prime },{\tilde{b}}_{\pi (2)}^{\prime }\right),\left({\tilde{a}}_{3}^{\prime },{\tilde{b}}_{\pi (3)}^{\prime }\right),...,\left({\tilde{b}}_{\pi (8)}^{\prime },{\tilde{a}}_{8}^{\prime }\right),\left({\tilde{b}}_{\pi (9)}^{\prime },{\tilde{a}}_{9}^{\prime }\right)\right\}. \end{array}$$

Aggregating ${\tilde{M}}_{AB}$ and ${\tilde{M}}_{AB}^{\prime }$, we obtain $\tilde{M}{\tilde{M}}_{AB}^{\prime }$.

$$\begin{array}{c} \tilde{M}{\tilde{M}}_{AB}^{\prime }=\left\{\left({\tilde{a}}_{1},{\tilde{b}}_{\pi (1)}\right),\left({\tilde{a}}_{2},{\tilde{b}}_{\pi (2)}\right),{\tilde{(a}}_{3},{\tilde{b}}_{\pi (3)}\right),...,\left({\tilde{b}}_{\pi (8)},{\tilde{a}}_{8}\right),\left({\tilde{b}}_{\pi (9)},{\tilde{a}}_{9}\right),\left({\tilde{a}}_{1}^{\prime },{\tilde{b}}_{\pi (1)}^{\prime }\right),\left({\tilde{a}}_{2}^{\prime },{\tilde{b}}_{\pi (2)}^{\prime }\right),\\ \left({\tilde{a}}_{3}^{\prime },{\tilde{b}}_{\pi (3)}^{\prime }\right),...,\left({\tilde{b}}_{\pi (8)}^{\prime },{\tilde{a}}_{8}^{\prime }\right),\left({\tilde{b}}_{\pi (9)}^{\prime },{\tilde{a}}_{9}^{\prime }\right)\}. \end{array}$$

Given ${\tilde{M}}_{AB}$ and ${\tilde{M}}_{AB}^{\prime }$, we have the following values for the *sign test* statistic:

$$\begin{array}{c} S_{{\tilde{M}}_{AB}}\left(A\right)=S_{{\tilde{M^{\prime }}}_{AB}}\left(A\right)=7,\;S_{{\tilde{M}}_{AB}}\left(B\right)=S_{{\tilde{M}}_{AB}^{\prime }}\left(B\right)=2;\\ S_{\tilde{M}{\tilde{M}}_{AB}^{\prime }}\left(A\right)=14\;S_{\tilde{M}{\tilde{M}}_{AB}^{\prime }}\left(B\right)=4. \end{array}$$

From our previous analysis of *M*_*AB*_ and $MM_{AB}^{\prime }$, we have that:

$$\begin{array}{c} P\left(S_{M_{AB}}\geq 8\right)=0.0098;P\left(S_{MM_{AB}^{\prime }}\geq 16\right)=0.000484. \end{array}$$

With respect to Theorem 2, then, we have that

$$\begin{array}{c} \frac{P(S_{MM_{AB}^{\prime }}\geq 16)}{P(S_{M_{AB}}\geq 8)}=\frac{0.000484}{0.0098}\approx 0.0494. \end{array}$$

We also have that

$$\begin{array}{c} \frac{P(S_{\tilde{M}{\tilde{M}}_{AB}^{\prime }}\geq 14)}{P(S_{{\tilde{M}}_{AB}}\geq 7)}=\frac{0.009211}{0.04779}\approx 0.193. \end{array}$$

This result illustrates the (general) property shown in Theorem 2. Namely, the *sign test* *p-value* exhibits a greater proportional decrease when replicated as the *p-value* for the *sign test* of the primitive data decreases (i.e., for more significant *sign test* results of the primitive data). Not only does the *sign test* exhibit *consistency upon aggregation*. *Sign test* results are more strongly reinforced in aggregation—in terms of proportional *p-value* decrease—as the original *sign test p-value* (of the primitive data) decreases.

Returning to *F*_*AB*_, *M*_*AB*_, $FF_{AB}^{\prime }$, and $MM_{AB}^{\prime }$, we iterate this aggregation to combine three and then four instances of the primitive ordinal data as follows.

#### The case of 3 Ordinal Replicates Aggregated

We now take

$$\begin{array}{c} FF^{\prime }F_{AB}^{\prime \prime }=a_{1},a_{2},a_{3},a_{4},a_{5},a_{6},a_{7},a_{8},b_{1},b_{2},b_{3},b_{4},b_{5},b_{6},b_{7},b_{8},b_{9},a_{1}^{\prime },a_{2}^{\prime },a_{3}^{\prime },a_{4}^{\prime },a_{5}^{\prime },a_{6}^{\prime },a_{7}^{\prime },a_{8}^{\prime },b_{1}^{\prime },b_{2}^{\prime },b_{3}^{\prime },b_{4}^{\prime },b_{5}^{\prime },b_{6}^{\prime },b_{7}^{\prime },b_{8}^{\prime },b_{9}^{\prime },F_{AB}^{\prime \prime },a_{9}^{\prime },a_{9} \end{array}$$

where $F_{AB}^{\prime \prime }=a_{1}^{\prime \prime },a_{2}^{\prime \prime },a_{3}^{\prime \prime },a_{4}^{\prime \prime },a_{5}^{\prime \prime },a_{6}^{\prime \prime },a_{7}^{\prime \prime },a_{8}^{\prime \prime },b_{1}^{\prime \prime },b_{2}^{\prime \prime },b_{3}^{\prime \prime },b_{4}^{\prime \prime },b_{5}^{\prime \prime },b_{6}^{\prime \prime },b_{7}^{\prime \prime },b_{8}^{\prime \prime },b_{9}^{\prime \prime },a_{9}^{\prime \prime }$ is ordinally equivalent to *F*_*AB*_ and therefore to $F_{AB}^{\prime }$, and

$$MM^{\prime }M_{AB}^{\prime \prime }=\{\left(a_{1},b_{\pi (1)}\right),\left(a_{2},b_{\pi (2)}\right),\left(a_{3},b_{\pi (3)}\right),...,\left(b_{\pi (9)},a_{9}\right),\left(a_{1}^{\prime },b_{\pi (1)}^{\prime }\right),\left(a_{2}^{\prime },b_{\pi (2)}^{\prime }\right),\left(a_{3}^{\prime },b_{\pi (3)}^{\prime }\right),...,\left(b_{\pi (9)}^{\prime },a_{9}^{\prime }\right),\left(a_{1}^{\prime \prime },b_{\pi (1)}^{\prime \prime }\right),\left(a_{2}^{\prime \prime },b_{\pi (2)}^{\prime \prime }\right),\left(a_{3}^{\prime \prime },b_{\pi (3)}^{\prime \prime }\right),...,\left(b_{\pi (9)}^{\prime \prime },a_{9}^{\prime \prime }\right)\}.$$

For this case, the *WMW test p-value* is 0.122, and the *sign test p-value* is 0.000027. The *WMW test p-value* has risen once again in level of aggregation, whereas the *sign test p-value* has once again fallen.

#### The case of 4 Ordinal Replicates Aggregated

Now take

$$FF^{\prime }F^{\prime \prime }F_{AB}^{\prime \prime \prime }=a_{1},a_{2},...,a_{7},a_{8},b_{1},b_{2},...,b_{8},b_{9},a_{1}^{\prime },a_{2}^{\prime },...,a_{7}^{\prime },a_{8}^{\prime },b_{1}^{\prime },b_{2}^{\prime },,b_{8}^{\prime },b_{9}^{\prime },a_{1}^{\prime \prime },a_{2}^{\prime \prime },...,a_{7}^{\prime \prime },a_{8}^{\prime \prime },b_{1}^{\prime \prime },b_{2}^{\prime \prime },...,b_{8}^{\prime \prime },b_{9}^{\prime \prime },F_{AB}^{\prime \prime \prime },a_{9}^{\prime \prime },a_{9}^{\prime },a_{9}\;$$

where $F_{AB}^{\prime \prime \prime }=a_{1}^{\prime \prime \prime },a_{2}^{\prime \prime \prime },a_{3}^{\prime \prime \prime },a_{4}^{\prime \prime \prime },a_{5}^{\prime \prime \prime },a_{6}^{\prime \prime \prime },a_{7}^{\prime \prime \prime },a_{8}^{\prime \prime \prime },b_{1}^{\prime \prime \prime },b_{2}^{\prime \prime \prime },b_{3}^{\prime \prime \prime },b_{4}^{\prime \prime \prime },b_{5}^{\prime \prime \prime },b_{6}^{\prime \prime \prime },b_{7}^{\prime \prime \prime },b_{8}^{\prime \prime \prime },b_{9}^{\prime \prime \prime },a_{9}^{\prime \prime \prime }$ is ordinally equivalent to *F*_*AB*_, $F_{AB}^{\prime }$, and therefore to $F_{AB}^{\prime \prime }$, and

$$\begin{array}{ccc} MM^{\prime }M^{\prime \prime }M_{AB}^{\prime \prime \prime } & = & \{\left(a_{1},b_{\pi (1)}\right),\left(a_{2},b_{\pi (2)}\right),\left(a_{3},b_{\pi (3)}\right),...,\left(b_{\pi (9)},a_{9}\right),\left(a_{1}^{\prime },b_{\pi (1)}^{\prime }\right),\left(a_{2}^{\prime },b_{\pi (2)}^{\prime }\right),\left(a_{3}^{\prime },b_{\pi (3)}^{\prime }\right),\\ & & ...,\left(b_{\pi (9)}^{\prime },a_{9}^{\prime }\right),\left(a_{1}^{\prime \prime },b_{\pi (1)}^{\prime \prime }\right),\left(a_{2}^{\prime \prime },b_{\pi (2)}^{\prime \prime }\right),\left(a_{3}^{\prime \prime },b_{\pi (3)}^{\prime \prime }\right),...,\left(b_{\pi (9)}^{\prime \prime },a_{9}^{\prime \prime }\right),\left(a_{1}^{\prime \prime \prime },b_{\pi (1)}^{\prime \prime \prime }\right),\\ & & \left(\left(a_{2}^{\prime \prime \prime },b_{\pi (2)}^{\prime \prime \prime }\right),a_{3}^{\prime \prime \prime },b_{\pi (3)}^{\prime \prime \prime }\right),...,\left(b_{\pi (9)}^{\prime \prime \prime },a_{9}^{\prime \prime \prime }\right)\}. \end{array}$$

For this case, the *WMW test p-value* is 0.209, and the *sign test p-value* is less than 0.00001. The *WMW test p-value* has risen once again in level of aggregation, whereas the *sign test p-value* has once again fallen. The overall significance testing results for the primitive and aggregated data are represented in Table 1.

**Table 1:** Application 1 statistical test *p-values*.

Test	Data Set	*p-value*
*WMW Rank Sum*	*F*_*AB*_	0.0027
*WMW Rank Sum*	$FF_{AB}^{\prime }$	0.0438
*WMW Rank Sum*	$FF^{\prime }F_{AB}^{\prime \prime }$	0.122
*WMW Rank Sum*	$FF^{\prime }F^{\prime \prime }F_{AB}^{\prime \prime \prime }$	0.209
*Sign Test for Matched Pairs*	*M*_*AB*_	0.0098
*Sign Test for Matched Pairs*	$MM_{AB}^{\prime }$	0.000484
*Sign Test for Matched Pairs*	$MM^{\prime }M_{AB}^{\prime \prime }$	0.000027
*Sign Test for Matched Pairs*	$MM^{\prime }M^{\prime \prime }M_{AB}^{\prime \prime \prime }$	<0.00001

#### Why does the WMW test display inconsistency upon aggregation?

Boudreau et al. [52] explore the social choice properties of rank sum scoring. They find that rank sum scoring approaches may invite various, related (social choice) paradoxes when a group with a superior rank sum “falls behind” (i.e., has fewer elements represented) at some point in the outcome sequence. In the application, group *A* has a superior rank sum in the primitive outcome sequence. However, group *A* also possesses the weakest overall element in the primitive outcome sequence. This makes group *A*’s significant rank superiority in the primitive outcome sequence vulnerable upon aggregation. In the aggregated data, if sufficiently many elements position after element *b*_9_ but before element *a*_9_ (as did occur), this particular interposition disproportionately inflates group *A*’s rank sum score. Such an outcome is not possible for matched pairs ordinal data aggregation. In that case, pairs remain assigned such that the primitive ordinal data and its replicate(s) reinforce one another.

## 5 (Empirical) Application 2: The aggregation paradox in ranking cross-country running team performance

As in Hammond [51] and Boudreau et al. [52], we use the setting of team cross country races to understand aggregation properties of ordinal data. In a cross country running meet, rank sum scoring is used as the official scoring methodology to assign team rankings. Boudreau et al. [52] describe the scoring methodology in detail:

In cross country running, for example, teams are compared on the performances of individual runners in a race; the outcome sequence is the overall ranking as to how runners finish. Rank sum scoring means that each group (team) receives a number of points equal to an element-wise ranking in the outcome sequence (1 for 1st place, 2 for 2nd, and so on), and comparison of groups is based on the sum of their element scores: groups with lower scores rank above those with higher scores [p. 220].

In the present application, we have identified two high school cross country programs for comparison: Carmel High School (Carmel, Indiana) and Fishers High School (Fishers, Indiana). We consider these two schools because their respective Varsity (Boys) teams competed against one another during the 2016 season, as did their respective Junior Varsity (Boys) teams. The two meets—the Varsity Indiana High School Athletic Association Noblesville Sectional meet and the Junior Varsity Hamilton County meet—occurred on the same running course (White River Elementary School Course; Noblesville, Indiana) such that the aggregation of the two data sets is valid (i.e., runners across meets faced the same basic performance elements). Moreover, Carmel’s Varsity and Junior Varsity teams won each respective meet by the very same convincing margin when rank sum scored against Fishers without the consideration of third-party teams (as in the *WMW test*). That is to say, respective pairwise rank sum scores were the same for each school in each race. Lastly, this particular setting was chosen because both the Varsity and Junior Varsity meets have been publicly recorded on *Athletic.net* (from which we obtained the data). It is typically difficult to find publicly-available Junior Varsity meet results. The results of these two cross country meets can be found at https://www.athletic.net/CrossCountry/meet/119851/results/481308 and at https://www.athletic.net/CrossCountry/Results/Meet.aspx?Meet=119845&show=all respectively.

This setting provides something of a natural experiment by which we can compare the two Cross Country programs at different levels of aggregation (i.e., a comparison of Varsity teams, a comparison of Junior Varsity teams, and an aggregated comparison of the two programs across the levels of competition) under the same basic competitive conditions. From the finishing time results, we obtain the observed primitive outcome sequence for the Varsity meet as

$$\begin{array}{c} F_{V}\left(C,S\right)=c_{1},c_{2},s_{1},c_{3},c_{4},c_{5},s_{2},c_{6},s_{3},c_{7},s_{4},s_{5},s_{6},s_{7}, \end{array}$$

where *F*_*V*_(*C*, *S*) symbolizes the observed Varsity race outcome sequence for our two Varsity teams, *C* = {*c*_1_, *c*_2_, *c*_3_, *c*_4_, *c*_5_, *c*_6_, *c*_7_} is a set representing the 7-element (7-runner) Carmel Varsity team, and *S* = {*s*_1_, *s*_2_, *s*_3_, *s*_4_, *s*_5_, *s*_6_, *s*_7_} is a set representing the 7-element (7-runner) Fishers Varsity team. The resulting rank sum scores are

$$\begin{array}{c} R_{F_{V}}\left(C\right)=36\;\;\;\;\text{and}\;\;\;\;\;R_{F_{V}}\left(S\right)=69, \end{array}$$

and the upper-tail *WMW rank sum p-value* for this sample difference, $P(R_{F_{V}}\geq 69|n=14)$, is equal to 0.019. In a *WMW rank sum test* of the Varsity data, then, we conclude that *C*≻_*α* = 0.025_ *S*. At the *α* = 0.025 significance level or any higher *α*-level, that is, we conclude that Carmel’s 2016 Varsity team was faster than Fisher’s 2016 Varsity team.

For the Junior Varsity meets, the observed race outcome sequence is given by

$$\begin{array}{c} F_{JV}^{\prime }\left(C^{\prime },S^{\prime }\right)=c_{1}^{\prime },c_{2}^{\prime },c_{3}^{\prime },s_{1}^{\prime },c_{4}^{\prime },s_{2}^{\prime },c_{5}^{\prime },c_{6}^{\prime },s_{3}^{\prime },c_{7}^{\prime },s_{4}^{\prime },s_{5}^{\prime },s_{6}^{\prime },s_{7}^{\prime }, \end{array}$$

where $F_{JV}^{\prime }\left(C^{\prime },S^{\prime }\right)$ symbolizes the observed race outcome sequence for our two Junior Varsity teams in the Junior Varsity race, $C^{\prime }=\left\{c_{1}^{\prime },c_{2}^{\prime },c_{3}^{\prime },c_{4}^{\prime },c_{5}^{\prime },c_{6}^{\prime },c_{7}^{\prime }\right\}$ is a set representing the 7-element (7-runner) Carmel Junior Varsity team, and $S^{\prime }=\left\{s_{1}^{\prime },s_{2}^{\prime },s_{3}^{\prime },s_{4}^{\prime },s_{5}^{\prime },s_{6}^{\prime },s_{7}^{\prime }\right\}$ is a set representing the 7-element (7-runner) Fishers Junior Varsity team. This leads to rank sums

$$\begin{array}{c} R_{JV}^{\prime }\left(C\right)=36\;\;\;\;\text{and}\;\;\;\;\;R_{JV}^{\prime }\left(S\right)=69. \end{array}$$

We see that the rank sum scores for the two teams are the same for the Varsity and Junior Varsity meets. Then, the upper-tail *WMW rank sum p-value* for the Junior Varsity sample difference, $P(R_{F_{JV}}\geq 69|n=14)$, is also equal to 0.019. In a *WMW rank sum test* of the Junior Varsity data, then, we conclude that *C*≻_*α* = 0.025_ *S*. At the *α* = 0.025 significance level or at any higher *α*-level, that is, we conclude that Carmel’s 2016 Junior Varsity team was significantly higher in quality than Fisher’s 2016 Junior Varsity team.

One might ponder the utility of ranking teams at the Junior Varsity level. Junior Varsity competition often provides a leading indicator of a program’s future strength. Further, the quality of a Junior Varsity team can indicate program depth and robustness against injury. We might therefore expect a strong overall program to be strong at each level.

Using finishing times, we pool the two outcome sequences to create the aggregated sequence

$$\begin{array}{c} FF_{VJV}^{\prime }=c_{1},c_{2},f_{1},c_{3},c_{4},c_{5},f_{2},c_{6},f_{3},c_{7},f_{4},f_{5},f_{6},f_{7},c_{1}^{\prime },c_{2}^{\prime },c_{3}^{\prime },f_{1}^{\prime },c_{4}^{\prime },f_{2}^{\prime },c_{5}^{\prime },c_{6}^{\prime },f_{3}^{\prime },c_{7}^{\prime },f_{4}^{\prime },f_{5}^{\prime },f_{6}^{\prime },f_{7}^{\prime } \end{array}$$

and obtain

$$\begin{array}{c} R_{VJV}^{\prime }\left(C\right)=170\;\;\;\;\text{and}\;\;\;\;\;R_{VJV}^{\prime }\left(F\right)=236. \end{array}$$

As with its constituent outcome sequences, $FF_{VJV}^{\prime }$ represents the true (observed) outcome sequence when the two meets (races) are pooled and runners are sequenced in ascending order of finishing time. Given the observed primitive race sequences, the pooled (aggregated) outcome sequence is simply the 14 Varsity elements (runners) in their primitive order followed by the 14 Junior Varsity elements. In other words, the first element in outcome sequence *F*_*JV*_ follows the final element in outcome sequence *F*_*V*_. Each meet represents a distinct level of competition by which these two cross country programs are ranked against one another.

Given that $R_{VJV}^{\prime }\left(C\right)=170$ and $R_{VJV}^{\prime }\left(F\right)=236$, the *WMW rank sum p-value* for this sample difference, $P(R_{VJV}^{\prime }\geq 236|n=28)$, is equal to 0.069. In a *WMW rank sum test* of the aggregated data, then, we conclude that (*C*∼_*α* = 0.025_ *S*), where “∼_*α* = 0.025_” reads “ranks indifferently to at the *α* = 0.025 significance level.” In fact, we also find that (*C*∼_*α* = 0.05_ *S*). At the *α* = 0.05 significance level or any smaller *α*-level, that is, we conclude from the aggregated data that Carmel’s 2016 Boys Cross Country program was not significantly different from that of Fishers. We obtain this result despite finding significant evidence that each of Carmel’s Boys Cross Country (primitive or constituent) teams (i.e., the Varsity and Junior Varsity teams) separately ranks significantly higher than its respective counterpart team at Fishers High School. In other words, we find an empirical example of an aggregation paradox when comparing these two Cross Country programs. This aggregation paradox is fairly extreme. Not only does the test of aggregated data lose significance at the *α* = 0.025 level but also at the *α* = 0.05 level. Further, the *p-value* for the aggregated data is more than the sum of the *p-values* for the two constituent data sets!

Of course, there are alternatives in sport to rank sum scoring. For example, team tennis matches typically employ a methodology that is consistent with the *sign test* methodology. For a dual team tennis match, each team assigns an intra-squad rank to each player or set of players, and each player for a given team is match paired to play against the player on the opposing squad who possesses the same intra-squad rank. Matches then take place and a team winner is assigned based on majority rule aggregation of individual match results. We can apply such a scoring rule to the case of team cross country. More specifically, we can apply this *sign test* type team tennis scoring to the application data from the two high school cross country meets considered.

$$\begin{array}{c} M_{V}\left(C,S\right)=\{\left(c_{1},s_{1}\right),\left(c_{2},s_{2}\right),\left(c_{3},s_{3}),...,\left(c_{7},s_{7}\right)\right\}\\ M_{JV}^{\prime }\left(C,S\right)=\{\left(c_{1}^{\prime },s_{1}^{\prime }\right),\left(c_{2}^{\prime },s_{2}^{\prime }),\left(c_{3}^{\prime },s_{3}^{\prime }\right),...,\left(c_{7}^{\prime },s_{7}^{\prime }\right)\right\} \end{array}$$

In *sign test* type team tennis scoring, each Carmel runner defeated his assigned pair on the Fishers team (for each of the two competition levels). As such,

$$\begin{array}{c} S_{M_{V}}\left(C\right)=7\;S_{M_{V}}\left(S\right)=0\\ \;\;\;S_{M_{JV}^{\prime }}\left(C\right)=7\;S_{M_{JV}^{\prime }}\left(S\right)=0 \end{array}$$

Then, we have that $P\left(S_{M_{V}}\left(.\right)\geq 7|\frac{n}{2}=7\right)=P\left(S_{M_{JV}^{\prime }}\left(.\right)\geq 7|\frac{n}{2}=7\right)=0.004075$. By aggregating the two data sets, we obtain

$$\begin{array}{c} MM_{VJV}^{\prime }\left(C,S\right)=\left\{\left(c_{1},s_{1}\right),\left(c_{2},s_{2}\right),\left(c_{3},s_{3}\right),...,\left(c_{7},s_{7}\right),\left(c_{1}^{\prime },s_{1}^{\prime }\right),\left(c_{2}^{\prime },s_{2}^{\prime }\right),\left(c_{3}^{\prime },s_{3}^{\prime }\right),...,\left(c_{7}^{\prime },s_{7}^{\prime }\right)\right\} \end{array}$$

and

$$\begin{array}{c} S_{MM_{VJV}^{\prime }}\left(C\right)=14\;S_{MM_{VJV}^{\prime }}\left(S\right)=0 \end{array}$$

Then, we have that $P\left(S_{MM_{VJV}^{\prime }}\left(.\right)\geq 14|\frac{n}{2}=7\right)=P\left(S_{MM_{VJV}^{\prime }}\left(.\right)\geq 14|\frac{n}{2}=14\right)=0.000091$. The *sign test p-value* diminishes in level of aggregation for the empirical application. Whereas the results of the *WMW test* are *inconsistent upon aggregation* in this empirical application, we again observe an instance of the general *consistency upon aggregation* quality of the *sign test*.

## 6 Application 3: Aggregation properties of the binomial test for equality of dependent proportions

McNemar’s test for the equality of proportions of matched pairs considers the discordant pairs with responses (Yes, No) and (No, Yes). Let *n* be the total number of discordant pairs, and *X* be the number of discordant pairs with response (Yes, No). Then the test statistic is given by $T=\left\{|X-\left(n-X\right)|-1\right\}/\sqrt{n}$, and the null hypothesis is rejected if *T*^2^ is too large (see for example, Rosner ([53], p. 375)). Under the null hypothesis, conditioned on *n* *X* has a Binomial distribution with parameters *n* and *p* = 1/2. When *n* is small (say under 20) the exact Binomial distribution of *X* is used ([53], p. 377) to compute the *p* value and the null hypothesis of equality of proportions is rejected when *X* is too small or large. When *n* is large, chi-square approximation with 1 degree of freedom is used and the upper tail probability associated with observed *T*^2^ is used as the *p* value. Thus, when number of discordant pairs in two data sets are equal, say *n* each, we can use Theorem 2 to establish strong form consistency upon aggregation of the test. When the number of discordant pairs vary, at least when the *n*_*i*_ are large, (11) can be used to establish consistency upon aggregation.

## 7 Application 4: Computation of *Yule-Simpson Paradox* for a case of rank sum scoring

Here, we consider a case of rank sum scoring in which two groups, each with two elements, are compared. The outcome of the comparison is a 4-element sequence (e.g., *a*, *b*, *b*, *a*). There are 4!/(2!2!) = 6 such sequences. For each sequence, we replicate that same sequence and pool the two ordinally-identical data sets to create every possible pooled sequence of eight elements for the two groups that preserves the within-sample ordering for each original sequence. For example *a*, *b*, *b*, *a* can be replicated and pooled with its replicate, *a*′, *b*′, *b*′, *a*′ to create *a*′, *b*′, *b*′, *a*′, *a*, *b*, *b*, *a* or, alternatively, *a*, *b*, *b*, *a*′, *a*, *b*′, *b*′, *a*′. For each of the six sequences in this case, there are 70 possible poolings. This can be easily verified via a decision tree. Essentially, there are up to 5 “bins” in which to place elements of the replicate data set into the original data set, but the number of bins for a given element is constrained because one cannot re-order the original, constituent sequences. A decision tree shows that there are $[\Sigma_{k=1}^{5}\Sigma_{i=1}^{k}i]=70$ possible poolings for each original sequence. Across all 6 original sequences, there are 70 ⋅ 6 = 420 possible poolings. We find that 72 of these poolings (17.14 percent) result in an instance of *Yule-Simpson Paradox* of one of the following type: *R*_*c*_(*A*)≤*R*_*c*_(*B*) but *R*_*p*_(*A*)>*R*_*p*_(*B*) or *R*_*c*_(*A*)≥*R*_*c*_(*B*) but *R*_*p*_(*A*)<*R*_*p*_(*B*). That is, one group has at least as low a rank sum score as the other in the constituent data set, but this is not the case in the pooled data set. This result suggests that instances of *Yule-Simpson Paradox* are fairly regularly occurring for small sample cases. Qualitatively, the result is in line with previous small sample results on *Yule-Simpson Paradox* incidence for other tests (see [28]; [54]).

## 8 Conclusion

In this work, we have used social choice theory and non-parametric statistical theory to examine the *Yule-Simpson Aggregation Paradox* as it applies to non-parametric statistical tests. As discussed, the *Paradox* relates to a broader literature concerning the effectiveness of information aggregation mechanisms. Herein, we have shown that the *sign test* for matched pairs exhibits general *consistency upon aggregation*. This is the first non-parametric test for which this property has been demonstrated generally, while several non-parametric tests have been shown in the prior literature to *not* possess this property. This result illustrates that there exists a non-parametric statistical test for which *consistency upon aggregation* is *possible*. In the present work, moreover, we find paired matching of ordinal data (across groups) to be important toward the result of general *consistency*. We prove an additional result as to the nature of *sign test consistency*. Namely, the *sign test p-value* exhibits a greater proportional decrease in aggregation as the original (primitive data *sign test*) *p-value* itself becomes smaller. In aggregation, then, we expect a greater proportional *p-value* decline given significant *sign test* results for the constituent, primitive data (than for insignificant *sign test* results). This represents something of a *strong form consistency* result, whereby data aggregation reinforces significant *sign test* results to a greater (proportional) degree.

Incorporating a generalized form of data aggregation, we also generate preliminary evidence that the *sign test* possesses a generalized form of *consistency upon aggregation*, in which the primitive data sets have varying sample sizes. For tractability, *n-aggregation* has been incorporated as a standard approach in examining the *Yule-Simpson Paradox* for ordinal data. However, analysis of the generalized, *n*_*i*_ − *aggregation* form is an important consideration. Often, data of different sample sizes are pooled (e.g., in the case of many longitudinal and unbalanced panel data sets). While we believe that the result holds for the *n*_*i*_ − *aggregation* problem as well, our proof is incomplete; we can formally claim the consistency only for large samples (see Theorem/Conjecture 3). With the *n* − *aggregation* form, we proved the consistency for any *n* by directly comparing *P*(*X*_*n*_ ≥ *k*) and *P*(*X*_*N*_ ≥ *rk*) where *N* = *rn*. This approach does not seem to work when the *n*_*i*_ are unequal. We focused on the *p* = 1/2 case, as our interest was on the *p* − *value*, or on the property of the test under the typical null hypothesis. If one is interested in the power properties, we need similar results for *p* ≠ 1/2. Using the normal approximation, we can conclude that the inequality stated in (11) holds for *c* > *p* when *n*_1_ and *n*_2_ are large and sufficiently apart.

We further incorporate a generic application that demonstrates tests of the same (aggregated) data by the *sign test* and by the *WMW rank sum test*, respectively. In the example, the *WMW test* results exhibit *inconsistency upon aggregation*. *Sign test* results demonstrate (the predicted) *consistency upon aggregation*, however. In the application, we further verify that the sign test results exhibit “strong form consistency” in aggregation.

An empirical application obtained from Indiana Boys High School Cross Country running data demonstrates a real-world application exhibiting both *rank sum inconsistency* and *sign test* *consistency*. Future work might evaluate alternative matched pairs style non-parametric tests to determine if there is a family of such tests possessing *consistency upon aggregation*. Indeed, the property is of central importance. As all data sets can be viewed as a potential aggregation of primitive data sets (i.e., from the power set of the aggregated set of data), *consistency* tells us generally whether a given statistical test evaluates data in an unambiguous manner.
